# Effectiveness of Streptococcus Pneumoniae Urinary Antigen Testing in Decreasing Mortality of COVID-19 Co-Infected Patients: A Clinical Investigation

**DOI:** 10.3390/medicina56110572

**Published:** 2020-10-29

**Authors:** Antonio Desai, Orazio Giuseppe Santonocito, Giuseppe Caltagirone, Maria Kogan, Federica Ghetti, Ilaria Donadoni, Francesca Porro, Victor Savevski, Dario Poretti, Michele Ciccarelli, Filippo Martinelli Boneschi, Antonio Voza

**Affiliations:** 1 Emergency Department, Humanitas Clinical and Research Center, IRCCS, 20089 Milan, Italy; giuseppe.caltagirone@humanitas.it (G.C.); maria.kogan@humanitas.it (M.K.); federica.ghetti@humanitas.it (F.G.); ilaria.donadoni@humanitas.it (I.D.); francesca.porro@humanitas.it (F.P.); antonio.voza@humanitas.it (A.V.); 2 Department of Biomedical Sciences, Humanitas University, 20090 Pieve Emanuele, Italy; 3 Department of Diagnostic and Interventional Radiology, Humanitas Clinical and Research Center IRCCS, Rozzano, 20089 Milan, Italy; giuseppe.santonocito@humanitas.it (O.G.S.); dario.poretti@humanitas.it (D.P.); 4 Artificial Intelligence Center, Humanitas Clinical and Research Center, IRCCS, 20089 Milan, Italy; victor.savevski@humanitas.it; 5 Pneumology Department, Humanitas Clinical and Research Center, IRCCS, 20089 Milan, Italy; michele.ciccarelli@humanitas.it; 6 Dino Ferrari Centre, Neuroscience Section, Department of Pathophysiology and Transplantation (DEPT), University of Milan, 20122 Milan, Italy; filippo.martinelli71@gmail.com; 7 Neurology Unit and MS Centre, Fondazione IRCCS Ca’ Granda Ospedale Maggiore Policlinico, 20122 Milan, Italy

**Keywords:** antibodies, bacterial infection 2, COVID-19, SARS-CoV-2, *Streptococcus pneumoniae*

## Abstract

Background and objectives: *Streptococcus pneumoniae* urinary antigen (u-Ag) testing has recently gained attention in the early diagnosis of severe and critical acute respiratory syndrome coronavirus-2/pneumococcal co-infection. The aim of this study is to assess the effectiveness of *Streptococcus pneumoniae* u-Ag testing in coronavirus disease 2019 (COVID-19) patients, in order to assess whether pneumococcal co-infection is associated with different mortality rate and hospital stay in these patients. Materials and Methods: Charts, protocols, mortality, and hospitalization data of a consecutive series of COVID-19 patients admitted to a tertiary hospital in northern Italy during COVID-19 outbreak were retrospectively reviewed. All patients underwent *Streptococcus pneumoniae* u-Ag testing to detect an underlying pneumococcal co-infection. Covid19+/u-Ag+ and Covid19+/u-Ag- patients were compared in terms of overall survival and length of hospital stay using chi-square test and survival analysis. Results: Out of 575 patients with documented pneumonia, 13% screened positive for the u-Ag test. All u-Ag+ patients underwent treatment with Ceftriaxone and Azithromycin or Levofloxacin. Lopinavir/Ritonavir or Darunavir/Cobicistat were added in 44 patients, and hydroxychloroquine and low-molecular-weight heparin (LMWH) in 47 and 33 patients, respectively. All u-Ag+ patients were hospitalized. Mortality was 15.4% and 25.9% in u-Ag+ and u-Ag- patients, respectively (*p* = 0.09). Survival analysis showed a better prognosis, albeit not significant, in u-Ag+ patients. Median hospital stay did not differ among groups (10 vs. 9 days, *p* = 0.71). Conclusions: The routine use of Streptococcus pneumoniae u-Ag testing helped to better target antibiotic therapy with a final trend of reduction in mortality of u-Ag+ COVID-19 patients having a concomitant pneumococcal infection. Randomized trials on larger cohorts are necessary in order to draw definitive conclusion.

## 1. Introduction

Bacterial co-infection is known to be associated with higher mortality in the setting of viral and parasitic diseases. This is due to severe inhibition of the host’s immune system as well as an increased resistance to the antibacterial therapy [1,2,3]. The devastating effects of a bacterial pneumonia in determining serious and fatal outcomes are not a novelty, as have been previously described in detail in association to other pandemic and non-pandemic viruses [4,5,6,7,8,9]. Even in the context of a co-colonization, bacteria may become pathogenic when a viral-induced immunosuppression occurs. As for coronavirus disease 2019 (COVID-19), limited data regarding the rate of concomitant infection have been portrayed in recent studies, reporting a broad range of variability (ranging from 2.1% to 94.2%) of co-infections and superinfections, most commonly bacterial [10,11,12,13,14,15,16,17]. Co-infection has been proven to have the highest incidence in severe-to-critical patients, in particular in those aged 15–44 and within 1–4 days of onset of COVID-19 disease [14]. Co-infection may also hinder an accurate diagnosis of severe acute respiratory syndrome coronavirus-2 (SARS-CoV-2) infection [11,15,16]. *Streptococcus pneumoniae* was found to be the most common pathogen associated with co-infection in these patients, followed by *Klebsiella pneumoniae* and *Haemophilus influenzae* [10,11,12,13,14]. Accordingly, an early diagnosis of *S. pneumoniae* and SARS-CoV-2 co-infection has been hypothesized to be an important factor in order prevent a series of potentially deadly complications in COVID-19 patients. The prophylactic use of pneumococcal conjugate and polysaccharide vaccines have even been recommended by some authors to decrease the risk of co-infection by the most common pathogens [14,18,19]. Nowadays, the presence of *S. pneumoniae* infection can be assessed by *S. pneumoniae* urinary antigen (u-Ag) testing, a non-invasive, inexpensive test based on an immunochromatographic technique, and its rapid result can guide the choice of antibiotic regimen in the emergency department (ED) settings [20,21,22,23]. A recent study by Kim and colleagues strongly advised the routine testing for non–SARS-CoV-2 respiratory pathogens during the COVID-19 pandemic in order to improve the clinical management of these patients, ultimately providing clinical benefits [11].

The aim of this retrospective cross-sectional study is to assess the effectiveness of the routine use of *S. pneumoniae* u-Ag testing in decreasing the overall mortality and in-hospital length of stay of COVID-19 patients with a pneumococcal co-infection. 

## 2. Materials and Methods

All subjects gave their informed consent for inclusion before they participated in the study. The study was conducted in accordance with the Declaration of Helsinki, and the protocol was approved by the Ethics Committee of Humanitas Clinical and Research Center, a tertiary referral Hospital in Milan located in Lombardy Region in northern Italy. Medical charts, management protocols, therapies, and overall morbidity and mortality data of a consecutive series of patients admitted to the ED from April to September 2020 with a diagnosis of SARS-CoV-2 infection were retrospectively reviewed. Data were analyzed in order to assess the role of *S. pneumoniae* u-Ag testing in the diagnosis of pneumococcal co-infection, as well as assessment of therapeutic protocols and the outcome in terms of length of hospital stay and survival analysis. 

### Outcome Measures

We compared Covid19+/u-Ag+ patients with Covid19+/u-Ag- in terms of overall survival and in-hospital length of stay. Differences in categorical responses between the two groups and in the pattern of computed tomography (CT) distribution were analyzed using a chi-square test performed on commercially available software (IBM SPSS software version 26.0). A survival analysis was also performed. 

Data were reported according to the Strengthening the Reporting of Observational Study in Epidemiology statement [24].

## 3. Results

### 3.1. Criteria for Suspicion of SARS-CoV-2 Infection and Emergency Management

Presumed diagnosis was based on predetermined signs and symptoms suggestive of infection, namely fever, dry cough, sore throat, fatigue, dyspnea, shortness of breath, chest pain, diarrhea, rash or discoloration of fingers or toes, and loss of sense of taste or smell. In patients presenting with previously described signs and symptoms, mean oxygen saturation (SpO2) and arterial oxygen partial pressure to fractional inspired oxygen ratio (P/F ratio) were measured. Reverse transcriptase–polymerase chain reaction (RT-PCR) for SARS-CoV-2 was routinely performed on nasopharyngeal swab along with *S. pneumoniae* u-Ag testing. Diagnosis of SARS-CoV-2 infection on RT-PCR was based on the World Health Organization interim guidance [25]. Chest CT scans were performed in all patients and independently reviewed by two expert radiologists, who classified the results in four different categories. Pediatric patients were excluded from the cohort, as those were managed in a dedicated pediatric ED. In case of confirmed SARS-CoV-2 infection and documented pneumonia, an antibiotic regimen was administered, and antiviral therapy was added to the regimen in case symptoms presented inferior to a 7-day period. In addition, hydroxychloroquine was used as an immune modulator in COVID-19 patients and administered only after the electrocardiographic exclusion of arrhythmias. Low-molecular-weight heparin (LMWH) was also prophylactically prescribed. None of the patients were treated with steroids. 

### 3.2. Antibiotic Protocol

Antibiotic protocol included a combination of Ceftriaxone 2 g IM/IV twice daily for 7–10 days and Azithromycin 500 mg PO once daily for 3 consecutive days. When one or both drugs were contraindicated, Levofloxacin stand-alone 750 mg PO/IV once daily for 5 days was administered. In case of acute bacterial exacerbation of an already known chronic bronchitis, Levofloxacin 500 mg PO/IV once daily for ≥7 days was the choice. Doses were adjusted for renal insufficiency case-by-case based on creatinine and calculated glomerular filtration rate. 

### 3.3. Antiviral Protocol

The off-label antiviral protocol entailed Lopinavir/Ritonavir 200 mg/100 mg PO twice daily or Darunavir/Cobicistat 800/150 mg PO once daily, both administered until clinical improvement. 

### 3.4. Hydroxychloroquine

Hydroxychloroquine 200 mg PO twice a day was also started by default in all patients immediately after COVID-19 diagnosis.

### 3.5. Low Molecular Weight Heparin

Enoxaparin or Nadroparin calcium were used for prophylaxis once daily with a dosage adjusted based on weight and renal function. 

### 3.6. Indications for Hospitalization

The need for hospitalization was evaluated case-by-case, considering multiple factors such as age, clinical status, respiratory parameters, and comorbidities. CURB65, which is a clinical algorithm validated for predicting mortality in community acquired pneumonia (CAP) and infection of any site and pneumonia severity index (PSI)/Pneumonia Patient Outcome Research Team (PORT) scores were used [26,27].

### 3.7. Incidence of SARS-CoV-2 Infection and SARS-CoV-2 and Pneumococcal Co-Infection

A total of 1247 consecutive cases of suspected SARS-CoV-2 infection were admitted to the ED, 575 of whom resulted positive for the virus (46%). *S. pneumoniae* u-Ag testing was performed in 536 patients (93%) of confirmed COVID-19 cases. Sixty-eight patients (13%) were diagnosed with co-infection (Covid19+/u-Ag+). Characteristics of patients with confirmed coinfection included an average age of 62.9 years (SD: 12.8), of whom 47 patients were >60 years old (69.1%) and male/female ratio of 1. Among Covid19+/u-Ag+ patients, we identified a lower number of males compared to u-Ag- (50% vs. 68.4%; *p* = 0.003). Most common comorbidities were hypertension (35.5%), cardiovascular (25%), respiratory (11.3%), and malignancy (14.5%). Clinical features of Covid19+/u-Ag+ patients included fever in 60 (88%), cough in 41 (60%), dyspnea in 34 (50%), and diarrhea in 10 (15%). Mean SpO2 was 91.8%, with an underlying respiratory alkalosis in 74% of cases. P/F ratio was >300 in 41 patients (60%), between 300 and 200 in 20 patients (30%), and <200 in 7 (10%) cases.

### 3.8. Chest CT Scan Findings

None of COVID-19+/u-Ag- patients presented focal opacities on chest CT scan in comparison with 3 COVID-19+/u-Ag+ group (*p* = 0.001). Conversely, four main CT patterns were detected in COVID-19+/u-Ag+ group as follows: normal (8.8%), ground glass opacification (GGO) (67.6%), GGO and multifocal consolidative opacities (19.2%), and focal opacities (4.4%). On a second chest CT scan, performed in 47 patients at an average interval of 35.2 days ± from the first CT scan, the proportion of normal CT scans increased to 36.2%, GGO decreased (34%), and GGO and multifocal consolidative opacities increased to 29.8%. Among COVID-19+/u-Ag+, a lower mortality was seen in the group with solely GGO as compared to the other groups (8.8% vs. 21.7%; *p* = 0.07). Among patients with an initially normal CT scan, two patients had a worsening clinical course resulting in hospital mortality: the first was an 82-year-old male, in this case the second CT scan, performed 39 days later, showed GGO and multi-lobar involvement, whereas the second patient was a 38-year-old female who died 7 days after the hospitalization.

### 3.9. Therapeutic Algorithms and Clinical Course

Antibiotic treatment was given to all patients, whereas antiviral protocol was implemented in 45 u-Ag+ patients (65.7%). Hydroxychloroquine and Enoxaparin, or alternatively Nadroparin, were administered in 47 (70.1%) and 33 patients (80.7%), respectively. COVID-19+/u-Ag+ group had a lower percentage of patients who underwent treatment with Hydroxychloroquine (70.1% vs. 80.7%; *p* = 0.04). Eight patients (12%) required supplementary oxygen, four of whom (50%) have been subjected to orotracheal intubation performed in the ED. All COVID-19+/u-Ag+ patients were hospitalized. Transfer to an Intensive Care Unit was necessary in 14.7% of cases.

### 3.10. Overall Survival

**Figure 1:**
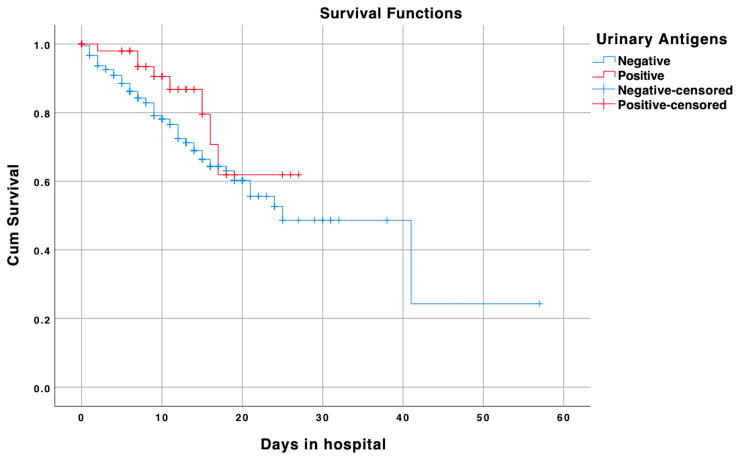
Survival analysis of Covid19+/u-Ag+ (red line) and Covid19+/u-Ag- (blue line) groups after emergency department admission. Censored cases are also shown for either groups: positive censored cases represent the end of follow-up of COVID-19 patients with positive urinary antigens, while negative censored cases represent the end of follow-up of COVID-19 patients with negative urinary antigens.

Overall mortality was 24.6%. Mortality was lower in Covid19+/u-Ag+ patients (15.4% vs. 25.9%; *p* = 0.09), and survival analysis confirmed a better prognosis in the prior group when compared to Covid19+/u-Ag- patients. Nevertheless, the data were not significant (log-rank test: *p* = 0.09; 75% percentile of survival: 12 days for Covid19+/u-Ag-, 16 days for Covid19+/u-Ag+) (Figure 1). 

### 3.11. In-Hospital Length of Stay

Median in-hospital stay was 10 days (interquartile range (IQR), 1–27 in COVID-19+/u-Ag+ patients, and 9 days in the COVID-19+/u-Ag- group (IQR 1–57, *p* = 0.71). Table 1 summarizes the overall data of the patients’ cohort (Table 1).

## 4. Discussion

In the current study we retrospectively reviewed a cohort of patients consecutively admitted to a tertiary hospital located in Northern Italy during the COVID-19 pandemic, with the aim to assess whether the early detection of SARS-CoV-2 and pneumococcal co-infection by *S. pneumoniae* u-Ag testing in ED setting is associated with a difference in mortality and length of hospital stay. Based on our findings, none of the previous were significantly influenced by the test. 

Bacterial co-infection has been reported to have a lower rate in COVID-19 patients as compared to other respiratory viral infections; the underlying factors for this finding are still largely unknown [28,29,30,31,32]. Nevertheless, SARS-CoV-2 and *S. pneumoniae* co- or super-infection has been reported to be an emerging source of concern, as it is associated with a worse or even fatal outcome in these patients [11,33,34,35]. The presence of a co-infection at hospital admission may mask the diagnosis of COVID-19. Lai and colleagues stressed that laboratory and imaging findings alone cannot help to distinguish co-infection from COVID-19, and that newly developed syndromic multiplex panels incorporating SARS-CoV-2 are necessary in order to improve early detection of co-infection [34]. Zahariadis and colleagues reported that diagnostic assays established for the most common pulmonary pathogens have several limitations, and the ruling out of SARS-CoV-2 by ruling in another pulmonary pathogen represents a non-negligible risk [35]. Notably, in 2004, Beadling and colleagues clarified that bacterial co- or super-infection of viral acute infections affects the outcome negatively through a severe alteration of the immune response; these include impaired natural killer cells and macrophage functions, co-activation of T cells, and lastly, hypersecretion of inflammatory cytokines. All of the previously depicted contribute to a lethal immunopathology [3]. Interestingly, similar cellular pathways have been reported in the pathogenesis of some central nervous system infections in which *S. pneumoniae* u-Ag has been thought as a potentially effective tool to detect an underlying pneumococcal infection in COVID-19 patients [36,37,38,39,40,41,42,43,44,45,46]. *Streptococcus pneumoniae* is an opportunistic Gram-positive pathogen that colonizes the mucosal surfaces of the human upper respiratory tract and establishes a commensal relationship with the host [47]. It is the most common pathogen responsible for community-acquired pneumonia [48]. In addition, in COVID-19, *S. pneumoniae* infection has been hypothesized to have an aggressive clinical course especially in some categories of patients considered at higher risk of mortality, namely those aged >65 years old, diabetics, and patients with a history of chronic alcohol abuse or immunosuppression [49,50]. Accordingly, an early diagnosis of pneumococcal infection may lead to tailored antibiotic regimen choice, which will allow optimal clinical management in high risk patients in addition to cost reduction. In our series, the incidence of *S. pneumoniae* infection detected with u-Ag testing was very similar to the one observed in a cohort of 1110 patients with clinical and radiographic evidence of pneumonia admitted to the ED of seven Utah hospitals in over two years, prior to COVID-19 pandemic [51]. Noteworthy, the evidence of pneumococcal infection ultimately led to the addition of Levofloxacin or Azithromycin to the treatment regimen of co-infected patients. Taking into consideration the unquestionable superiority of the most common invasive and non-invasive examinations in obtaining the microbiological evidence of pneumococcal infection, *S. pneumoniae* u-Ag testing has been associated with an average sensitivity and specificity of 70% and 95%, respectively [20,21,22,51,52,53]. This test is rapid, non-invasive, easily accessible, and repeatable. Recently, Charton and colleagues found that age, immunosuppressive factors, typical pneumococcal symptoms, and PSI scores correlate with a positive *S. pneumoniae* u-Ag test [53]. All the aforementioned factors led us to hypothesize that the routine use of *S.* pneumoniae u-Ag testing, implemented at our ED since the beginning of COVID-19 outbreak, might have affected the outcome and length of hospital stays in SARS-CoV-2/*S. pneumoniae* co-infected patients. Our data did not confirm this hypothesis; thus, we cannot recommend our management protocol in its integral form. Nevertheless, it is of relevance to consider the findings reported in our survival analysis, as they show a tendency for a better prognosis in Covid19+/u-Ag+ patients when compared to Covid19+/u-Ag-. Doubts do still exist by the authors about the implementation of a prospective and randomized trial involving a larger cohort of patients, which may lead to a different conclusion regarding the beneficial effect of *S. pneumoniae* co-infection on mortality rate and length of hospital stay. 

### Limitations

Limitations of this study include its retrospective nature, small sample size, and lack of blood or pleural fluid culture to confirm the diagnosis of *S. pneumoniae* co-infection. In part, these limitations are shared with other recent studies, as many were caused by the exceptional situation generated by the SARS-CoV-2 pandemic outbreak. It is worthwhile to mention the different and changing therapeutic protocols adopted during the management of COVID-19, in accordance to what divulged and recommended by the World Health Organization in the acute phase of the pandemic, which has unavoidably introduced biases in this and other similar studies. The timely treatment itself with targeted antibiotics in all patients having SARS-CoV-2 infection and documented pneumonia may have improved the prognosis of the co-infected patients, leading to an underestimation of the effectiveness of *S. pneumoniae* u-Ag test. 

## 5. Conclusions

The use of *S. pneumoniae* u-Ag testing, aimed at an early diagnosis of SARS-CoV-2 and *S. pneumoniae* co-infection, did not significantly affected the overall mortality and length of hospital stay of COVID-19 patients. Its routine use at hospital admission in the ED setting cannot therefore be recommended. However, in light of the multiple theoretical advantages coming from the ease, reliability, and cost-effectiveness of this test, as well as the trend toward statistical significance of reported data, further study and randomized trials are worthwhile to be implemented, since they may theoretically lead to different results. 

## Figures and Tables

**Table 1:** Clinical features of patients stratified by pneumococcal urinary antigen positivity.

Data	Positive (*n* = 68)	Negative (*n* = 468)	*p*-Value
Age, mean (SD), (range)	62.9 (12.8) (28–92)	65.1 (14.8) (27–93)	*p* = 0.19
Male, *n* (%)	34/68 (50%)	320/468 (68.4%)	*p* = 0.003
Comorbidities, median (IQR), (range)	1 (1) (0–7)	1 (1) (0–10)	*p* = 0.67
Antivirals, *n* (%)	44/67 (65.7%)	325/463 (70.2%)	*p* = 0.45
Antibiotics, *n* (%)	67/67 (100%)	427/464 (92%)	*p* = 0.02
Hydroxychloroquine, *n* (%)	47/67 (70.1%)	373/462 (80.7%)	*p* = 0.04
LMWH, *n* (%)	33/67 (49.3%)	188/461 (40.8%)	*p* = 0.18
Time to ED (days), median (IQR), (range)	5 (7) (0–25)	7 (7) (0–35)	*p* = 0.72
LOS in days, median (IQR), (range]	10 (7) (1–27)	9 (8) (1–57)	*p* = 0.71
Chest CT scan: normal	6 (8.8%)	21 (4.5%)	*p* < 0.001
GGO	46 (67.6%)	324 (69.2%)	
GGO and multifocal consolidative opacities	13 (19.2%)	123 (26.3%)	
Focal opacity	3 (4.4%)	0	
In-hospital death (*n*, %)	8/52 (15.4%)	98/378 (25.9%)	*p* = 0.09

LMWH: Low Molecular Weight Heparin; ED: Emergency Department; LOS: Length Of In-hospital Stay; GGO: Ground Glass Opacities.
